# Patient-specific silicone aortic model using 3D printing for Coselli graft planning in open thoracoabdominal aortic aneurysm repair

**DOI:** 10.1016/j.jvscit.2026.102399

**Published:** 2026-07-04

**Authors:** Nicolas Soro, Lucy Guazzo, Liam Georgeson, Nick Boyne, Jason Jenkins

**Affiliations:** a Herston Biofabrication Institute, Metro North Hospital and Health Services, Herston, Queensland, Australia; b Department of Vascular Surgery, The Royal Brisbane and Women's Hospital, Herston, Queensland, Australia

**Keywords:** 3D printing, Open aortic repair, Patient-specific model, Surgical simulation, Thoracoabdominal aortic aneurysm, Vascular surgery training

## Abstract

Open thoracoabdominal aortic aneurysm repair remains essential in selected patients but is infrequently performed, limiting opportunities for operative planning and training. We describe the use of a patient-specific compliant silicone aortic model, fabricated using three-dimensional printing and silicone molding, to support preoperative planning of open thoracoabdominal aneurysm repair using a Coselli graft. The model enabled realistic rehearsal of graft orientation, branch vessel reconstruction, and operative sequencing, with close correlation to intraoperative findings. This case demonstrates the feasibility of compliant physical modeling as a planning adjunct and highlights its potential value for surgical preparation and clinical training in complex open aortic surgery.

Despite advances in endovascular techniques, open repair remains preferable for selected patients with thoracoabdominal aortic aneurysm (TAAA), particularly younger individuals and those with suspected connective tissue disorders where long-term durability is paramount. Superior outcomes are demonstrated at high-volume centers^1^; however, in smaller population countries such as Australia, even centralized units may encounter limited cases.^2^

Given the morbidity associated with open TAAA repair, effective preoperative planning is critical. Computed tomography angiography (CTA) provides excellent anatomical detail but cannot convey three-dimensional (3D) spatial relationships, anastomotic geometry, or the dynamic changes that occur during surgical exposure, such as displacement of the left kidney.

Patient-specific physical models have been used across surgical specialties for education, device sizing, and procedural planning.^3^, ^4^, ^5^ In vascular surgery, applications have largely focused on aneurysm visualization and endovascular simulation, typically using rigid or semiflexible materials with simplified geometry.^6^^,^^7^ Their use in open TAAA repair remains limited. Park et al^8^ reported rigid 3D printed models for Coselli graft planning; however, rigid constructs do not permit realistic tissue interaction, including clamping or suturing. These limitations are particularly relevant for the off-the-shelf Coselli graft, where standardized branch configuration must be aligned with patient-specific visceral anatomy.

In this report, we describe a patient-specific compliant silicone aortic model fabricated using 3D printing and molding to support Coselli graft planning in open Crawford extent IV TAAA repair.

## Clinical case

A healthy 45-year-old male was referred to a tertiary vascular unit after self-detecting an abdominal pulsatile mass. He was asymptomatic with no family history of aortic aneurysm or dissection, no known connective tissue disorder, and clinical examination revealed no extravascular stigmata. CTA demonstrated a 5.9-cm Crawford extent IV TAAA extending from the celiac artery to the aortic bifurcation. The proximal common iliac arteries were ectatic but nonaneurysmal. A 19-mm right popliteal artery aneurysm was also identified.

Standard cardiorespiratory workup was undertaken, with unremarkable respiratory function tests, myocardial perfusion imaging, and transthoracic echocardiography. The presence of multisite aneurysmal disease in a young patient raised concern for an underlying connective tissue disorder. Genetic testing was planned but, given the aneurysm size, surgical repair was not deferred to await results. Following shared decision-making, an open surgical approach was favored over branched endovascular repair given the patient's young age, excellent functional status, and the concern for underlying aortopathy where long-term durability is paramount.

To facilitate preoperative planning, a patient-specific high-fidelity silicone aortic phantom was fabricated from the CTA data set using the technique described below.

Written informed consent was obtained from the patient for publication of this case and associated images.

## Model design and fabrication

**Fig 1:**
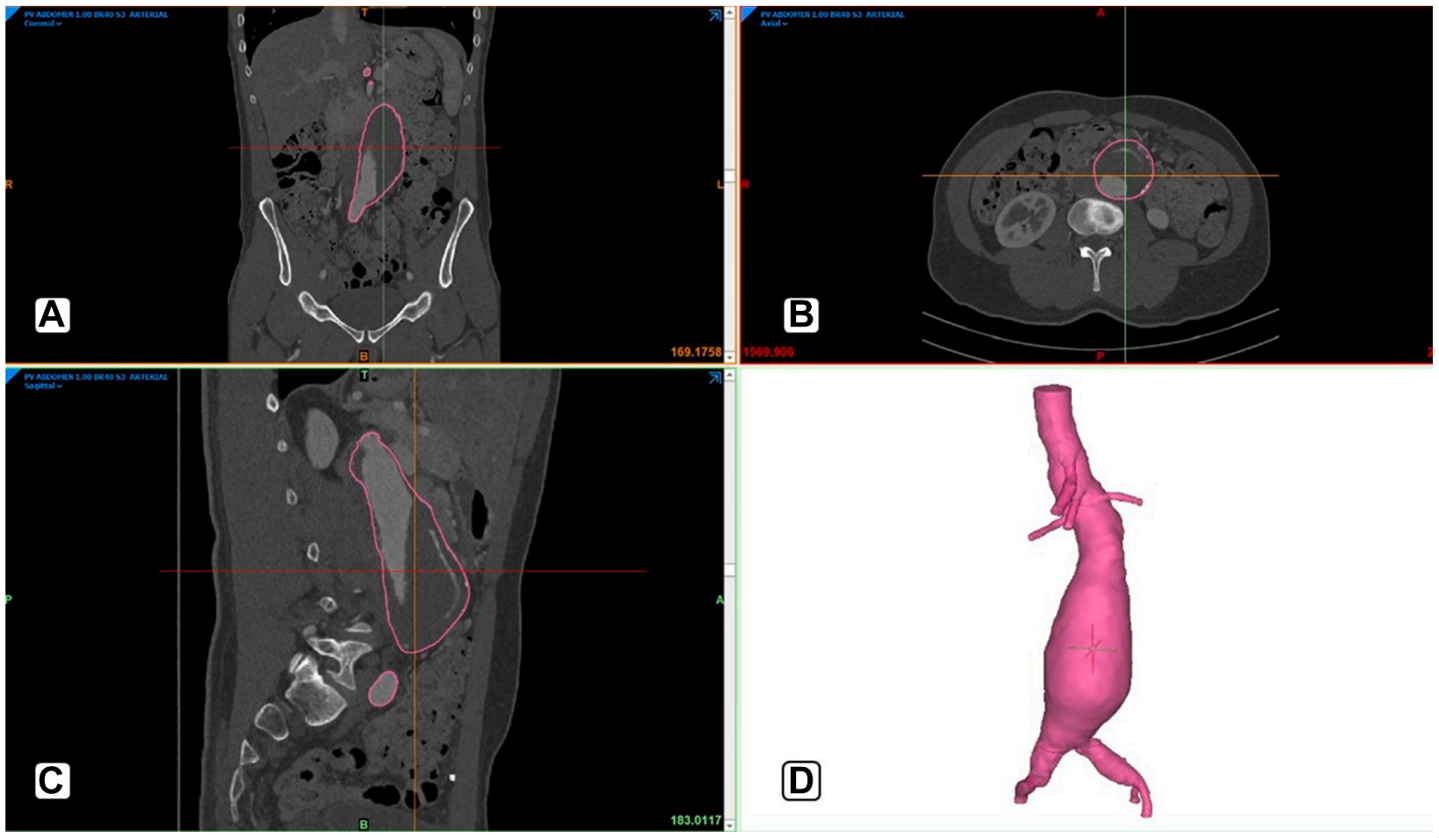
Preoperative computed tomography angiography (CTA) data set of the patient used for model generation: **(A)** coronal view; **(B)** axial view; **(C)** sagittal view; and **(D)** three-dimensional (3D) segmented reconstruction.

Patient-specific anatomy was derived from preoperative contrast-enhanced CTA (1-mm slice thickness; pixel spacing, 0.782 mm; Canon Aquilion ONE). CTA DICOM data were segmented using Mimics Innovation Suite (Materialise NV) to generate a 3D reconstruction of the anatomy (Fig 1). The reconstructed anatomy was imported into 3-matic (Materialise NV), where a multipart mold system with separable outer molds and an internal lumen core was designed for casting and demolding. The lumen core combined rigid PolyJet components with dissolvable fused deposition modeling-printed poly(vinyl alcohol) elements in constrained regions to facilitate removal after curing. Parting lines were positioned to minimize undercuts and allow extraction without damaging the cured model.

**Fig 2:**
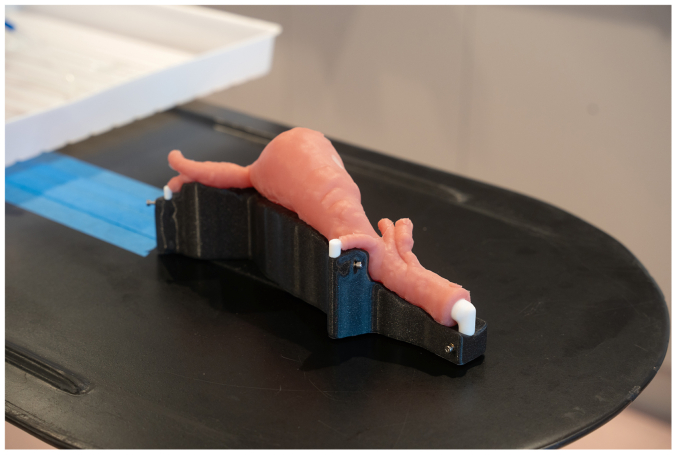
Patient-specific compliant silicone aortic model for open surgical simulation.

A uniform wall thickness of 2 mm was targeted throughout the model to provide a compliant yet suturable construct, based on previously reported vascular simulation approaches^9^, ^10^, ^11^ and iterative usability assessment with vascular surgeons during development. The outer mold components were fabricated using material jetting 3D printing.^12^ A two-part platinum-cure silicone elastomer (Shore 10A), selected to provide compliant handling and suturing characteristics consistent with previously reported studies,^9^, ^10^, ^11^ was degassed under vacuum prior to injection to minimize entrapped air. Following curing, the model underwent manual demolding, trimming, and visual inspection prior to simulation preparation (Fig 2).

Dimensional quality assurance was performed using fiducial reference measurements distributed throughout the mold geometry to assess distortion, shrinkage, and warping during fabrication and curing. Postfabrication measurements demonstrated dimensional deviation ranging from −0.22 mm to +1.04 mm relative to the computed tomography-derived geometry, remaining within a predefined engineering tolerance of ±2 mm considered acceptable for the targeted application.^6^^,^^13^

The complete workflow involved approximately 12 hours of active labor time, in addition to unattended additive manufacturing and silicone curing time. Under standard staffed business hours, the total turnaround time from imaging acquisition to completed model and rehearsal was approximately 7 days.

## Simulation

**Fig 3:**
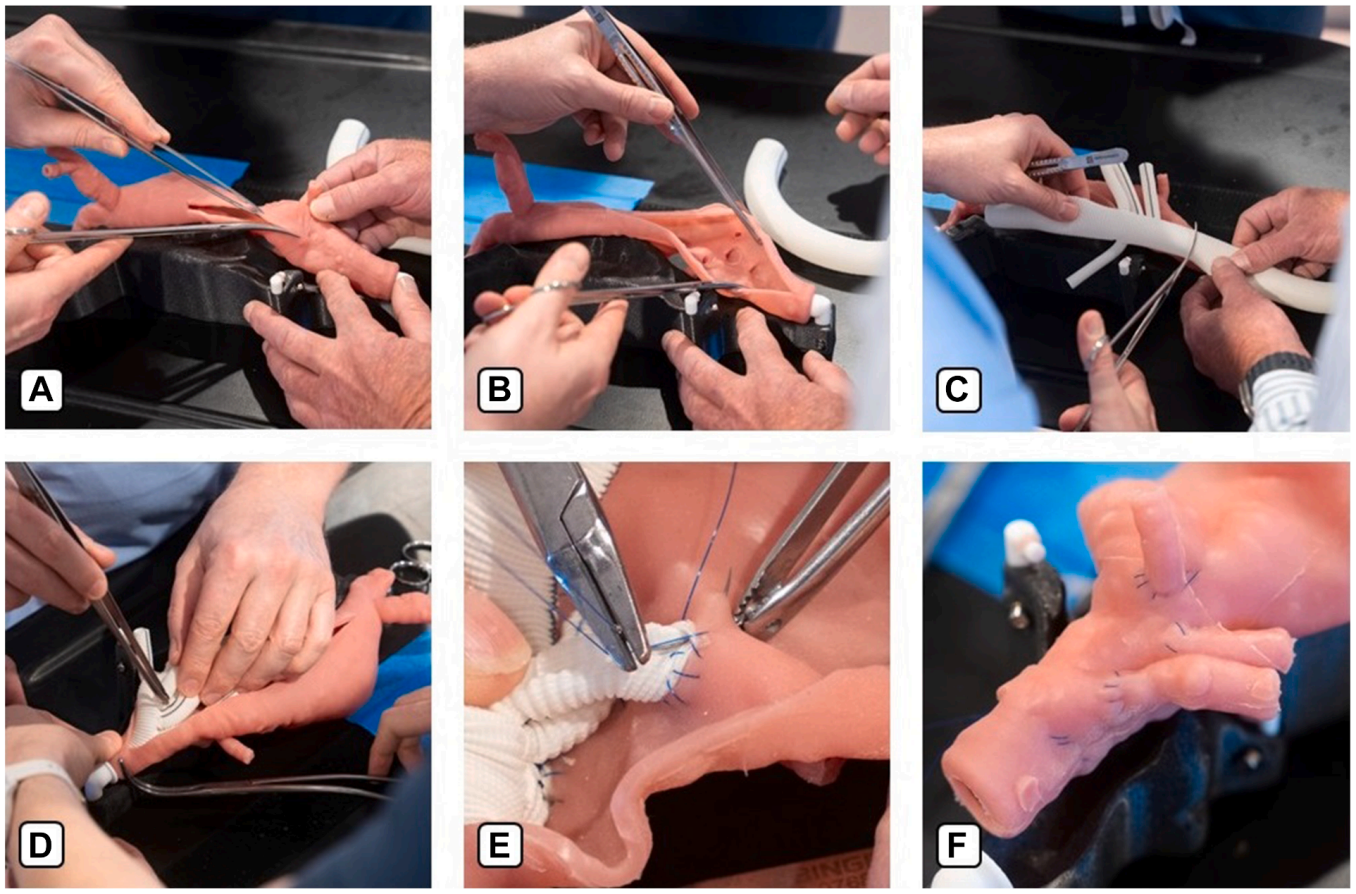
Open repair rehearsal on a patient-specific silicone aortic model, showing sutured anastomosis of a Coselli graft. **A,** Initial incision into the aneurysm sac. **(B)** Aneurysm sac opened to expose the aortic lumen. **(C)** Coselli graft trimming to appropriate length. **(D)** Determination of graft orientation and branch alignment within the model. **(E)** Example of side-branch anastomosis being sutured. **(F)** External view of the proximal anastomosis with completed suture lines.

The simulation was conducted 1 week prior to surgery at a tertiary biofabrication facility using an expired Coselli graft (Terumo Aortic). A single 45-minute preoperative rehearsal was performed by the lead surgeon, who completed a full reconstruction rehearsal, incising the aneurysm model and sequentially anastomosing the distal thoracic aorta, right renal artery, superior mesenteric artery, celiac artery, left renal artery, and aortic bifurcation according to the planned operative sequence (Fig 3).

Particular attention was given to graft orientation, branch configuration, and branch lengths, especially the left renal artery branch length, given anticipated displacement of the left kidney during exposure. Although kidney mobilization was not explicitly simulated, the model could be manipulated to assess its impact on graft orientation and branch length. Postreconstruction pressurization of the graft construct was not performed, as the primary objective of the rehearsal was assessment of procedural workflow and anatomical graft configuration during reconstruction.

## Intervention

A spinal drain was placed prophylactically the day prior to surgery. The patient was positioned in the left lateral decubitus position, and a thoracoabdominal incision was performed. The diaphragm was divided 2 cm from its margin, with marking sutures placed to facilitate subsequent repair. Cardiopulmonary bypass was established with split arterial cannulation via the distal thoracic aorta and femoral artery, with venous return via the femoral vein. The patient was cooled to 32 °C.

A 24-mm Coselli graft was used, with the proximal anastomosis fashioned 5 cm above the celiac origin using 3-0 Prolene. Sequential anastomoses were then performed to the right renal artery, superior mesenteric artery, celiac artery, and left renal artery, with a total visceral clamp time of 25 minutes for all four branch vessel anastomoses. An 8-mm side branch was anastomosed to the Coselli graft to perfuse a large lumbar artery at L2, reducing the risk of spinal cord ischemia.

**Fig 4:**
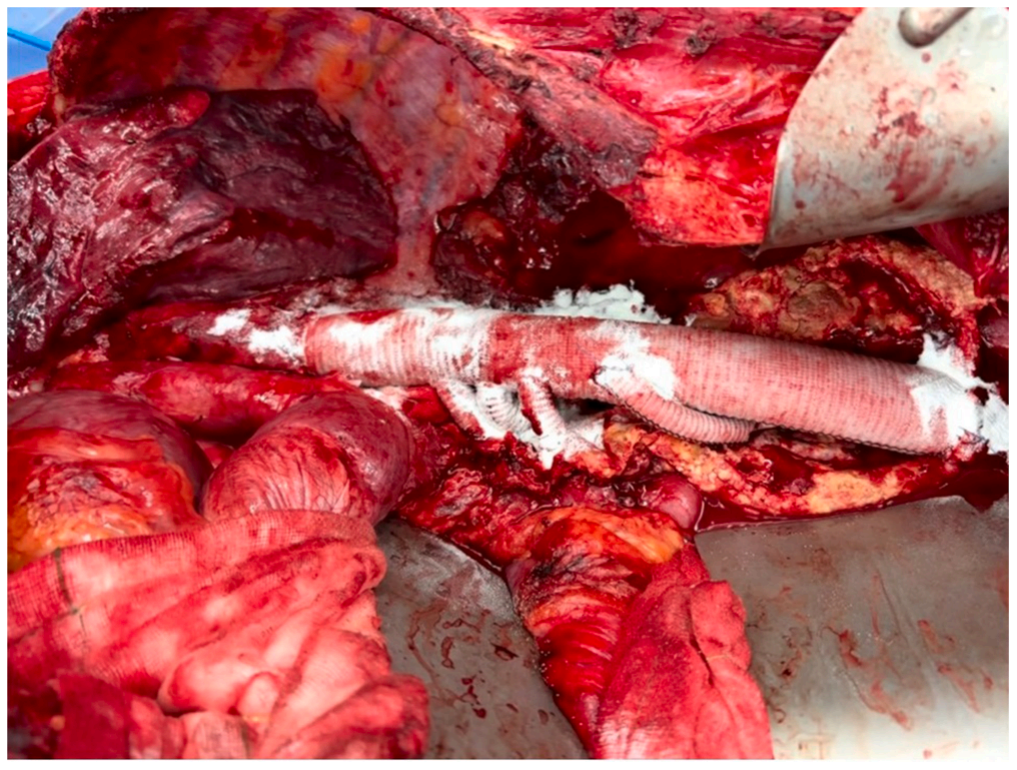
Intraoperative view after completion of open thoracoabdominal aortic aneurysm (TAAA) repair.

Intraoperative findings correlated well with the phantom simulation. The spatial relationships and graft configuration anticipated during rehearsal were consistent with those encountered at surgery (Fig 4). The branch vessel orientation and left renal artery length requirements predicted during simulation proved accurate, allowing confident graft positioning without intraoperative revision.

**Fig 5:**
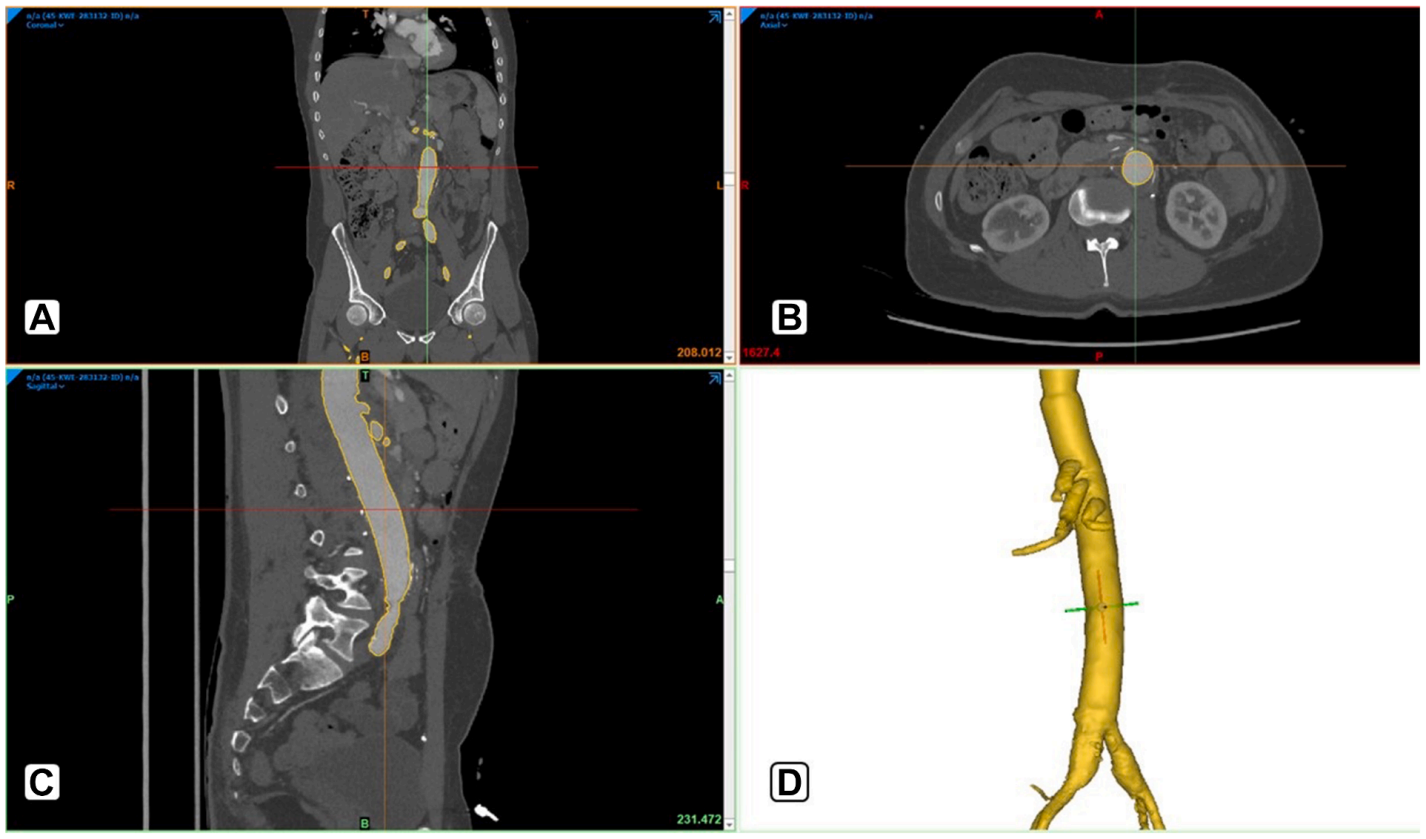
Postoperative computed tomography angiography (CTA) data set of the patient: **(A)** coronal view; **(B)** axial view; **(C)** sagittal view; and **(D)** three-dimensional (3D) segmented reconstruction.

The patient made an uncomplicated recovery with no evidence of malperfusion or renal injury. Follow-up CTA demonstrated satisfactory graft position with all branch vessels patent (Fig 5).

## Discussion

This case demonstrates how a compliant, patient-specific aortic model can support preoperative planning in complex open TAAA repair. Unlike imaging or rigid models, the silicone model permitted surgical interaction, including incision, suturing, and evaluation of graft handling and orientation, which are central to open reconstruction.

The principal value lies in aligning standardized branched graft geometry with patient-specific visceral anatomy and operative exposure. Rehearsal facilitated assessment of graft orientation, branch sequencing, and anticipated left renal branch length. These elements are difficult to appreciate from imaging alone. Not all intraoperative decisions are predictable during rehearsal; the addition of a lumbar side branch reflected physiological considerations beyond anatomical planning.

Model configuration also requires balancing realism with usability. While constrained mounting more closely replicates operative exposure, the lead surgeon detached the model from its base to optimize spatial understanding and efficiency. In contrast, maintaining thoracoabdominal constraints may be preferable for clinical training. This supports the development of a modular platform adaptable to both planning and educational objectives.

Limitations of this report include its single-case design and the absence of quantitative matching of material compliance to native aortic tissue.

The initial production cost of the patient-specific phantom was approximately Australian dollar $2427, largely driven by fabrication of the patient-specific molds. Once manufactured, the molds can be reused to produce additional silicone models of the same anatomy at substantially lower cost and reduced turnaround time, supporting repeated procedural rehearsal, training, and workshop applications.

Fabrication of patient-specific silicone models requires specialized expertise and infrastructure, which may limit routine use. However, selective application in anatomically complex cases, low-volume centers, and training settings may provide substantial benefit for rare, high-risk open aortic procedures.

## Conclusions

A patient-specific compliant silicone aortic model, fabricated using 3D printing, was successfully integrated into Coselli graft planning for open TAAA repair, with close correlation to intraoperative anatomy. This platform may serve as a practical adjunct for preoperative planning and selective training in complex open aortic surgery.

## Funding

This project was funded by 10.13039/100017511Metro North Health & Hospital Services.

## Declaration of generative AI and AI-assisted technologies in the writing process

During the preparation of this work, the authors used ChatGPT (OpenAI) in order to improve the clarity and readability of the manuscript text. After using this tool, the authors reviewed and edited the content as needed and take full responsibility for the content of the publication.

## Disclosures

None.
